# Emerging role of machine learning in light-matter interaction

**DOI:** 10.1038/s41377-019-0192-4

**Published:** 2019-09-11

**Authors:** Jiajia Zhou, Bolong Huang, Zheng Yan, Jean-Claude G. Bünzli

**Affiliations:** 1 Faculty of Science, Institute for Biomedical Materials and Devices, University of Technology, Sydney, NSW 2007 Australia; 2 Department of Applied Biology and Chemical Technology, The Hong Kong Polytechnic University, Hong Hum, Kowloon, Hong Kong SAR China; 3 Faculty of Engineering and IT, Centre for Artificial Intelligence, University of Technology, Sydney, NSW 2007 Australia; 4 Swiss Federal Institute of Technology, Lausanne (EPFL), ISIC, Lausanne, Switzerland

**Keywords:** Optical materials and structures, Microscopy

## Abstract

Machine learning has provided a huge wave of innovation in multiple fields, including computer vision, medical diagnosis, life sciences, molecular design, and instrumental development. This perspective focuses on the implementation of machine learning in dealing with light-matter interaction, which governs those fields involving materials discovery, optical characterizations, and photonics technologies. We highlight the role of machine learning in accelerating technology development and boosting scientific innovation in the aforementioned aspects. We provide future directions for advanced computing techniques via multidisciplinary efforts that can help to transform optical materials into imaging probes, information carriers and photonics devices.

## Artificial intelligence: ‘Black boxes’ shine light on optical materials

Machine learning algorithms are finding success at accelerating the screening of nanomaterials for optical devices including imaging probes and information storage platforms. Jiajia Zhou from the University of Technology Sydney in Australia and colleagues review how complex interactions between light and matter are being deciphered by training artificial intelligence software with data obtained from chemical and physical characterization techniques. For example, optical microscopy applications can now analyze images of 2D materials, such as graphene, and instantly report on their composition and thickness. Machine learning approaches have also boosted the data storage of silica nanostructures 40 percent higher than blue-ray discs by improving assessments of faint light signals originating below the diffraction limit. The authors caution that expert insights are still needed before ‘black boxes’ can generate materials predictions based solely on large data sets.

Recently, artificial intelligence has rapidly altered many aspects of modern society, enhancing the role of the machine in competing against different human roles. Machine learning (ML), as a subfield of artificial intelligence, is fast becoming an essential part of every industry, with an unprecedented ability to rapidly classify and predict patterns within data and discern unforeseen trends that are otherwise impossible for a human observer to identify. Almost every scientific field is benefitting from this powerful tool, be it for prediction, the generation of data or parameter estimation.

The machine learning algorithm called deep learning is becoming increasingly popular. It has a strong capability of finding latent data structures and classifying highly nonlinear datasets, attributes that are ideal for physical sciences and, more particularly, photonics. Taking advantage of embedded codes and algorithms from realistic theoretical calculations, deep learning can stretch their abilities to quickly evolve into a method for accelerating screening efficiency and the theoretical prediction of nanomaterials.

Research relevant to the light-matter interaction, facilitated by materials science, physics, and photonic technologies, is being promoted to a new level with the engagement of ML. This is evidenced by two new trends: one is the emerging intelligent photonic technologies; the other is embedding ML into physical and chemical sciences for insightful knowledge acquisition and novel fundamental discoveries.

## Intelligent photonics technology developments

“Intelligent” means that photonics technology with the upgrade functionality, which is enabled by machine learning techniques, is outperforming conventional photonics, which could be laborious, be time/cost consuming, and deliver limited performance.

Investigation of the light-matter interaction highly depends on optical microscopy, which allows the localized observation of an object in a controllable three-dimensional space and simultaneously translates the interaction into images or spectra. For further quantification of the image or spectra with correlated structural information, conventional studies rely on additional characterizations such as atomic force microscopy (AFM), transmission electron microscopy (TEM), and Raman spectroscopy. In the following two examples, we highlight the fact that ML shows great potential to develop new interpretation tools, resulting in user-friendly all-in-one technology.

### Structure-optical property correlated microscopy

**Fig. 1:**
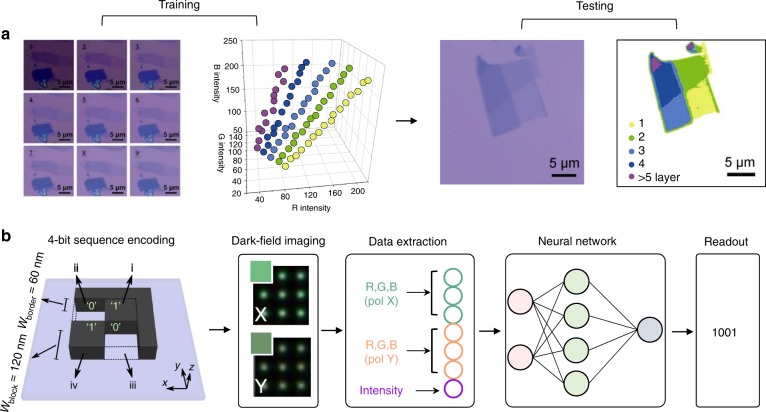
Examples of technology development with the assistance of machine learning. **a** Optical identification of 2D nanostructures in graphene and MoS_2_ using a support vector machine (SVM) algorithm^1^. The training set contains optical microscope photographs of graphene or MoS_2_ samples at different light intensities. Following the judgment based on atomic force microscopy (AFM) and Raman spectroscopy, the red-green-blue (RGB) database and SVM model of graphene or MoS_2_ samples (denoted as “training results”) are established after SVM analyses of the RGB data collected from the training set. Referring to the “training results”, graphene, MoS_2_, or heterostructures of these two materials can then be identified according to their optical microscope photographs (denoted as “testing results”). **b** Optical information read-out via the RGB values of microscopy images based on an artificial neural-network^12^. Within a “4-bit” nanostructure geometry, the digital information is encoded in the four silicon blocks (block: “1”, no block: “0”). The structure corresponds to the 4-bit digit “1001” (decimal “9”). The L-shaped sidewall is necessary to distinguish symmetric arrangements via polarized optical spectroscopy. Representative polarized (X-polarizations and Y-polarizations) filtered dark-field color images of representative 3 × 3 arrays of the “4-bit” digit structures are collected to extract the input feature, including R, G, and B values in both polarizations and the intensity value. A scheme of the fully connected artificial neural network is used for the RGB classification task and generates the “4-bit” digit output

Two-dimensional (2D) materials are extremely interesting in view of their interlayer coupling and carrier transfer behavior. The identification of the number of layers and their heterogeneity is a general requirement to facilitate device engineering and to reach an understanding regarding optical phenomena such as blinking and quantum emission. While the existing technologies, including STM, AFM, and Raman spectroscopy, can sense the thickness of 2D materials, they normally need to be performed separately from optical imaging and are not very conclusive. Hence, Lin and coworkers developed an optical microscope with self-customized ML software, which enables in situ wide-field identification of 2D nanostructures, as demonstrated for graphene and MoS_2_^1^. The training process established a database, and the machine learned the characteristic red-green-blue (RGB) information of the optical photographs, which was correlated to the layers of graphene or MoS_2_. During training, the ground-truth labels were the number of layers of the 2D samples provided by AFM characterization, which confirmed that the sample thickness was linked to different categories of RGB intensities and were further analyzed by the SVM algorithm to establish a training model. During testing, the false-color image visually displayed the number of layers and even impurities thanks to the classification made by the software based on logistic regression (Fig. 1a).

### Decoding technology beyond the diffraction limit

With the fast-growing development of nanotechnology, sophisticated structures and their miniaturizations have become mature tools. The resulting challenge is to accurately resolve faint optical signals from a limited number of atoms or molecules, which are very likely to be buried in noise. Moreover, the larger the extent of the nanostructure control is, the larger the database of the relevant optical parameters. In this case, ML can play the role of an unprecedented interpretation method, replacing conventional algorithms or manual operations. Recent works have employed ML to predict the bandgap^2–4^, Fermi level^5^, and optical spectra of nanophotonic particles and metasurfaces^6–10^. Moreover, Aharon and coworkers proved that deep learning can efficiently mitigate the adversarial effects of noise^11^.

The same situation occurs in optical data storage. Nanomaterials are leading a large movement towards optical storage methods with ultrahigh capacity, ultralong lifetime and ultralow energy consumption. However, their subtle features are difficult to read out accurately. High-density optical data storage is limited by light diffraction. To overcome this problem, Wiecha et al. developed a robust ML-based read-out technology enabling encoding multiple-bit information (Fig. 1b)^12^. Optical information of silica nanostructures is stored in scattering spectra or simply as RGB values from dark-field imaging. A neural network was trained to output 4-bit encoding values upon the input of both X-polarized and Y-polarized RGB datasets and the scattering intensities. The demonstrated information retrieval of the network was almost error-free, so that the network was further expanded to 9-bit encoding sequences, leading to a 40% higher storage capacity than that of blu-ray discs. This new encoding technology has enabled up to nine bits of information per diffraction-limited area, far beyond the one bit of information per area of traditional technology. The ability of neural networks to find hidden underlying patterns and form nonlinear decision boundaries within datasets makes them ideal for solving such problems.

As image-based ML algorithms belong to one of the most rapidly developing and mature fields of ML, it is interesting to test this technology on microscopy applications using algorithms spanning a wide range of complexity, from very simple pixel-based algorithms that look at the relationships between neighboring pixels and their values to more complex ones such as deep learning, which interprets the hidden information based on the input features from the image data. Exemplified work includes a report from Ozcan’s group, who developed a virtual staining technology for the histological analysis of tissues, in which the diffraction-limited input images are transformed into super-resolved images with the help of a convolutional neural network^13^.

## New ML tools for photonic materials discovery

**Fig. 2:**
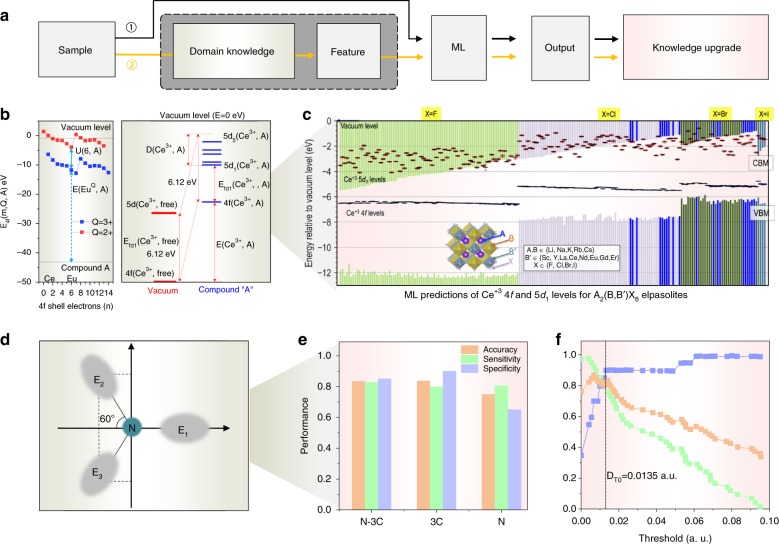
Demonstration of how machine learning helps in achieving a knowledge upgrade. **a** Flow chart highlighting the pathways leading to a knowledge upgrade with (② supervised training) or without (① unsupervised training) existing domain knowledge to extract meaningful features for ML. Upgraded knowledge is relative to existing knowledge in each domain, determined by the scientific problems we aim to solve. Two examples showcase the possible existing domain knowledge (**b, d**) and upgraded knowledge (**c, e, f**) for practical problems during ML. **b, c** Towards fast materials screening, the ML approach reveals key conditions for efficient Ce^3+^-activated scintillators and predicts good candidates^15^. **b** Left panel: 4 *f* vacuum-referred binding energies (VRBE) *E*_4*f*_ (*m*, *Q*, *A*) of the divalent (*Q* *=* 2 + ; red squares) and trivalent (*Q* *=* 3 + ; blue squares) lanthanide ions; *m* represents the number of electrons in the 4 *f* shell: *m* *=* *n* for *Q* *=* 3 + , and *m* *=* *n* *+* 1 for *Q* *=* 2 + ; “*A*” represents the chemical environment of the lanthanide ions. Right panel: Scheme depicting the changes in 4*f*-shell and 5*d*-shell electron binding energies in Ce^3+^ from a vacuum to a chemical environment *A*. **c**, Stacked-band scheme showing the DFT-computed relative valence and conduction band edges and ML-predicted VRBEs of an electron in the 4*f* and 5*d*_1_ levels of the Ce^3+^ activator for elpasolite compounds; known scintillators are highlighted with blue bars. **d**–**f** Towards the discovery of new aggregation-induced emission (AIE) materials, the ML approach predicts and understands the AIE effect based on quantum mechanics. **d** Triphenylamine (TPA) core: green circle represents central nitrogen atom, and gray ellipses represent adjacent carbons with charges E_i_^16^. **e** Classifiers trained by N-3C and 3 C show similar performance, while the single N yields the worst result. **f** Classification of the qualities of the parameter D at different threshold levels; the dot-dash line refers to the best threshold

The ‘black box’ principle of deep learning allows one to solve problems without a professional background or precise knowledge of the field. The ‘black box’ bypasses the intricate underlying physics and mechanisms, relying only on the training process and a large amount of data to carry out a task. To truly take advantage of artificial intelligence for human cognition and science advancement, the present tendency is to focus on solving a conventionally inextricable problem using new ML tools. The most challenging task is to choose the best input features for training the algorithm, which often requires in-depth knowledge of the problem, as well as of how the ML algorithm itself works. For cases where it is nearly impossible to engineer input features or have an intuition regarding the domain knowledge (the ‘black box’ remains unopen), ML provides opportunities to discover hidden relationships that humans are otherwise unable to perceive based on the unsupervised training from convolutional neural networks (CNNs) (Fig. 2a ⑴). By shedding light onto the black box of a deep neural network, after training and successful generalization, ML helps us further improve our intuition and domain knowledge through the alternative paths created by data-based inference. A possible method such as ‘transfer learning’ in deep learning could leverage the knowledge (features, weights, etc.) from previously trained models to train newer models and even tackle problems with less data for newer tasks^14^.

Path ⑵ in Fig. 2a shows the cases where it is possible to open the ‘black box’ directly by having a suitable background and fine scientific intuition for extracting effective features from experimental data via supervised training. Domain knowledge requires an in-depth mechanistic understanding of phenomena in addition to the phenomenological parameters of the studied materials, such as the size, shape, composition, and optical properties. New ML algorithms can subsequently determine the correlations between features and outputs. When the number of features is large and when they may include redundant members, pairwise relevance analysis allows one to reduce the number of features. This ability yields improved ML output performances and can reveal invaluable novel information. We present two examples pertaining to the connection between atomic or molecular characteristics and electronic behaviors.

The first case addresses Ce^3+^-activated scintillators, for which a strict requirement is that both the ground 4f^1^ and first excited 5d^1^ electronic configurations must lie within the bandgap of the host material. Pilania and coworkers developed a new ML model for the fast screening of numerous hosts, which correctly predicted that chloride and bromide elpasolites meet this condition and are good candidates (Fig. 2c)^15^. The generalized knowledge upgrade includes the following: a large 4f-vacuum band maximum (VBM) indicates a low hole capture probability leading to poor scintillation performance. The key background knowledge for performing this task is understanding the correlation between structural information and targeted parameters. The features selected as input for the ML model included density functional theory (DFT)-calculated bandgaps, ionization potentials, electronegativities, electron affinities, empirical radii, and atomic polarizations, among others. The ML model correctly predicted the two targeted parameters U (6, A), the energy difference between divalent and trivalent ions, and D (Ce^3+^, A), the crystal field splitting of the 5d^1^ configuration (Fig. 2b).

The second example addresses aggregation-induced emission (AIE) materials, which embody a unique optical property, standing in stark contrast to the more usual aggregation-induced quenching. Achieving targeted and controllable AIE is a real challenge, and most current syntheses are achieved through laborious trial and error because of our limited understanding of the luminescence-enhancing mechanism. Recently, Qiu and coworkers employed ML to predict the AIE activity of substituted triphenylamine (TPA) luminophores^16^. Inspired by previous experience, they chose the charge distribution of the TPA core (Fig. 2d), estimated by nature bond orbital analysis, as the input feature, and their ML model output a classification of the TPA derivatives as AIE active (P) or inactive (N). Compared with the training set, its predictions are classified as true P (TP), false N (FN), false P (FP), or true N (TN). Its performance is characterized by three parameters: accuracy (% of true answers), sensitivity, and specificity, where the latter two represent its ability to recognize P and N luminophores from the relevant classes. The model identified that AIE is sensitive to the charges on the three carbon atoms connected to the central nitrogen atom (Fig. 2e) and, more quantitatively, that AIE can be activated when the dipole moment (D) of the TPA core exceeds a given threshold (Fig. 2f).

## Future potential

Machine learning is starting to play a valuable role in building seamless connectivity between classical theories, scientific intuition and experiments from realistic optical responsive materials, but this approach is still in its infancy. The light-matter interaction is an intricate phenomenon, the description of which requires interactive work between materials science, physics, chemistry, optics, and engineering. There are many opportunities for machine learning to be invoked at different stages during the process of deciphering the mechanism of the light-matter interaction and while transforming optical materials into imaging probes, information carriers or optical devices. In turn, the machine learning paradigms must be adaptable to solve domain problems.

### Materials screening and discovery

Materials control light-matter interactions and determine the performances of optical devices. In turn, application-specific requirements constrain materials properties such as the electronic configuration, defect energetics, and structure and symmetry. Conventional trial-and-error methods used to discover ideal materials are time-consuming and resource-consuming. Fueled by the availability of extensive databases, materials discovery is now trying to incorporate machine learning to uncover hidden empirical knowledge from both successful and failed experiments^17,18^ or for extracting atomic or molecular features^19,20^. In several cases, machine learning models outperform traditional human-based strategies with great success^17^. However, this does not mean that future work can completely rely on ML. To further enhance the role of ML, precise knowledge of a scientific problem is indispensable for optimizing ML algorithms. Moreover, experimental work is always inescapable when training ML and improving its prediction capability, as noted by Oliynyk and coworkers^18^.

### Optical microscopy

Optical observation of an object at the microscale or nanoscale yields invaluable and indispensable information. Ideally, microscopy should be quick operating, have super-resolution and enable high throughput and multitasking. ML can upgrade current microscopy to a new level with significantly improved performances in resolution, throughput rate, and multitasking. Image analysis is crucial in extracting the sought after information, and specifically, ML algorithms have been reported to perform well in image processing. For example, deep learning enhances the imaging resolution^21,22^, accelerates the super-resolution imaging speed^23,24^, and performs image reconstruction^25,26^.

### Characterization of the structural-optical property correlation

A significant number of research projects aim at exploring and describing the light-matter interaction through characterization using ML. Most of them try to establish structure-property correlations in a fast and accurate manner through microscopic characterization and demonstrating the capability of deep learning in recognizing subtle information hidden in a structure^6,10,12^. With the target of device miniaturization, technology development is presently shifting from microscale to nanoscale operations^11^, where nanomaterials become key components. Future promising optical nanomaterials will need a carefully controlled design in terms of their optical stability, uniformity and diversity. To achieve this, single object characterization, which pushes the burden onto the microscopic resolution, throughput, and denoising approach, is essential. By leveraging the automated nature of ML, as well as its ability to rapidly find optimal solutions, high resolution, high throughput and signal-to-noise ratio are anticipated to be achieved in single nanoparticle characterization.

### Theoretical-calculation-guided optical materials exploration

The computation-guided design strategy has become a promising approach for materials studies based on high-throughput and accurate results. As a reliable theoretical method for probing the optical properties of both present and unknown materials, density functional theory (DFT) is able to supply initial recommendations for experimental trials based on a comprehensive understanding of optical processes, including the electronic state properties (such as the excitation energy and oscillator strength) and the dynamics of excited-state. These preliminary results will not only lower the experimental trial cost but also significantly contribute to the establishment of a broad-range optical materials database. Through the utilization of ML, the time-consuming issues of performing a theoretical calculation on complex systems will be further addressed, with the efficiency enhanced by several orders of magnitude. Given sufficiently large databases and sufficient domain knowledge, ML will achieve faster simulation and analysis of electronic structures, as well as the determination of unknown physical properties or applications by uncovering the hidden latent information within the experimental data^27^. The establishment of optical materials databases based on the collaboration between theoretical calculations and ML will accelerate unearthing the potential optical materials for a broad range of applications.

### Designing materials with targeted optical properties

Quantum optics, optical communications, photonics, and green energy production all require specific optical materials with improved performances. This means investigating the light-matter interaction within specific environmental constraints, targeting one or more vital characteristics. For example, constraining light resonance in a cavity is a requirement of laser materials, while the Q factor reflects the efficiency of the cavity with respect to its lasing capability. Recently, by using ML, Asano and coworkers reported that the Q factor of two-dimensional photonic crystal nanocavities could be optimized to 1.58 × 10^9^, more than two orders of magnitude higher than the base cavity^28^. Therefore, it seems that an increasing number of optical device fabrications and pilot trials will benefit from ML, increasing the success rate while reducing both the cost and fabrication time.

### Explainable machine learning for optical materials

The properties and characteristics of optical materials are governed by underlying physical principles. However, as most existing machine learning methods are black box in nature, the physics is ignored. The negative consequence of this situation is that researchers have difficulties interpreting the extent to which a cause and effect can be observed within a machine learning model. This indicates the significance of investigating explainable machine learning methods that will enable users to understand how the models make predictions and decisions. Several directions may be viable to achieve this goal. For example, Bayesian deep learning, which leverages the information on uncertainty, will be capable of telling what the models know and what they do not know^29^. It provides insights when the black box fails. Another paradigm for explainable machine learning is human-in-the-loop learning, which combines human and machine intelligence to create effective learning models based on a continuous feedback loop^30^. This means that expert knowledge will be fed into the model so that causal relations could be better constructed, and model bias will be avoided.

### Addressing uncertainty to capture fundamental optical properties

Machine learning relies on data, most of which are obtained through the experimentation of materials under various settings. The diverse combination of materials, research objectives, experimental settings, and operators will inevitably lead to inconsistency in the data distribution. This means that a model trained in one environment will fail to work properly in a new environment. To investigate this issue, one should pay attention to the impact of the concept drift of data^31^. In this scenario, transfer learning and drift learning may contribute to mitigating the problem. It is essential to investigate these learning paradigms in the area of light-matter interactions. This means that the understanding and characterization of the domains and data topology are critical. New criteria to evaluate the similarity or dissimilarity of problem domains should be developed. Correspondingly, although ML will undoubtedly bring photonic materials into a new age, novel learning methods and benchmark datasets are needed to keep it pointed in the right direction.
